# The paradox of growing technical capacities with low global governance: a review of Voluntary National Reviews’ SDG health-related indicators

**DOI:** 10.1186/s12992-024-01051-x

**Published:** 2024-06-21

**Authors:** Ana Luisa Jorge Martins, Rômulo Paes-Sousa

**Affiliations:** Health and Social Protection Policies Research Group, René Rachou Institute, Oswaldo Cruz Foundation (FIOCRUZ), Av. Augusto de Lima, 1715. Belo Horizonte, Belo Horizonte, Minas Gerais Brazil

**Keywords:** 2030 agenda, SDGs, Global health, Health-related indicators, Voluntary national review, Global health governance

## Abstract

**Background:**

This study delves into the States’ accountability for health-related Sustainable Development Goal (SDG) indicators from 2016 to 2020. An analysis of Voluntary National Reviews (VNR) is employed as an instrument to scrutinize the alignment of States’ indicators with the global indicator framework, shedding light on global health governance within the context of the 2030 Agenda and States’ strategic prioritization. A curation of 60 health-related indicators from 195 VNRs, produced during the aforementioned period, is organized into thematic groups.

**Results:**

Our results highlight a concerning discrepancy in the reporting frequency of various health-related themes. The findings reveal a paradoxical coexistence characterized by the concurrent strengthening and diminution of the global health governance articulated in the Agenda’s global health governance. This manifests in the increased utilization and consistency of health-related indicators over the study years, coupled with an emphasis on infectious diseases and child and maternal health indicators. Conversely, a discernible governance decline is evidenced by the inadequate representation of health-related indicators in VNRs, notably within the domains of universal health coverage and health system indicators. Furthermore, High-Income States exhibit diminished accountability.

**Conclusions:**

The VNRs unveil a paradox wherein burgeoning technical capacity coexists with governance deficits, a phenomenon attributable to both statistical capabilities and political preferences. The prevalent use of proxy indicators in VNRs oversimplifies the presentation of official indicators, thereby compromising the aspirational goal of pioneering statistical innovations for measuring intricate issues in the SDGs. In light of our conceptualization of the 2030 Agenda’s global health as a regime complex governance, we advocate for comprehensive investigations into each health regime cluster. This approach aims to unravel disputes, discern patterns, and elucidate States’ preferences concerning specific thematic areas. Functioning as an accountability mechanism for the Agenda’s governance, VNRs underscore States’ adaptability and short-term learning capabilities, offering valuable insights for identifying harmful goal prioritization. The discretionary nature of indicator selection by States in the VNRs, enabled by the Agenda’s proposition of a contextual adaptation of the SDGs and a blind eye to the guideline’s request to review all SDG indicators, highlights a critical flaw in the VNR as an accountability mechanism.

## Background

The 2030 Agenda stands as a pivotal achievement in the realm of international policy, notable for its inclusive construction for sustainable development, encapsulated in the formulation of 17 Sustainable Development Goals (SDGs) grounded in a holistic approach that seeks the integrality and interdependence of all goals [1, 2]. While considerable discourse has ensued regarding the design and breadth of global governance underpinning this Agenda, notably less attention has been given to the Voluntary National Review (VNR) – the mechanism through which national reporting occurs – and its consequential impact on governance dynamics [3, 4]. These VNRs, annual reports presented at the High-Level Political Forum on Sustainable Development (HLPF), serve as a crucial tool for monitoring and evaluating progress related to the 2030 Agenda. Our premise posits that the VNR constitutes a necessary instrument for sustaining the global governance architecture of the 2030 Agenda [4, 5]. Furthermore, we contend that the indicators showcased in VNRs offer a valuable lens through which to discern States’ positions within the broader landscape of global governance. This paper aims to scrutinize the health-related indicators featured in VNRs, examining their alignment with the official global methodology. Through this analysis, we aim to contribute substantively to the ongoing discussion surrounding the 2030 Agenda’s global health governance and shed light on States’ prioritization strategies within the overarching framework of the Agenda.

The VNRs, which function as mechanisms of accountability, serve the dual purpose of both enforcing [6] and measuring governance [5]. Nevertheless, the incorporation of global governance within the 2030 Agenda presents a formidable challenge, given that its goal-setting approach does not have a legal foundation or mechanisms of compliance to constrain the actions of participating States [7]. Pintér, Kok, and Almassy [5] emphasize the role of considering indicators and reporting mechanisms as integral components of the 2030 Agenda’s governance. Failure to address this aspect adequately could jeopardize the established decision-making process through the misguided application and alteration of metrics by individual States. They underscore the necessity of adopting reporting mechanisms characterized by a learning-adapting approach, incorporating both short- and long-term feedback. Such an approach is integral to tracking progress effectively and harmonizing the diverse array of actors and interests involved [5]. Furthermore, VNRs emerge as a valuable instrument for scrutinizing the diverse prioritizations made by States, as evident in their selection of indicators showcased in the reviews. This aspect is intrinsically tied to the global governance framework of the 2030 Agenda’s global governance [4].

The sweeping aspirations of the SDGs and the intricate interplay among their respective objectives pose a formidable challenge. This challenge is exacerbated by the absence of institutions and mechanisms capable of effectively defining and overseeing potential conflicts stemming from these interactions on a global scale. Consequently, this inadequacy is a potential impediment to the overarching global governance framework of the 2030 Agenda [8]. The specific health goal within this agenda, articulated as ‘Good Health and Well-Being’ (SDG 3), necessitates a synergistic approach with other health-related targets embedded in the broader framework. It positions health at the confluence of well-being and sustainability, underscoring the exigency of formulating novel arrangements [9–11]. The SDGs, therefore, present an opportune juncture to advance a contemporary paradigm of global health [12]. Over the past decade, a myriad of proposals have surfaced in the literature, seeking to reformulate the concept of global health in consonance with the SDGs. These include initiatives such as ‘One Health’ [13], ‘Planetary Health’ [14] and analogous propositions founded upon an expansive conceptualization of health and well-being [15, 16]. However, the discourse on global health grapples with significant imprecision, offering definitions that predominantly oscillate between confined micropolitical considerations and broader macropolitical perspectives [17]. The complexity intensifies when contemplating a pragmatic concept of global health governance that is simultaneously operationalizable at the national level and expansive enough to accommodate emerging paradigms in health design.

Within the academic discourse, a consensus regarding the prevailing structure of global governance within the framework of the 2030 Agenda remains elusive [8, 18, 19]. Antedating the emergence of this Agenda, endeavors were undertaken to devise governance structures that acknowledge interdependencies while preserving the distinctiveness inherent in various health-related issues. Fidler’s [20] theoretical framework on global health governance stands as a notable contribution, delving into the intricacies of social determinants of health, the nuanced nature of their relationships, and the associated challenges within the contemporary global health scene. We suggest that Fidler’s perspective exhibits a commendable breadth and adaptability requisite for accommodating the expanded conceptualization of health mandated by the 2030 Agenda. Fidler delineates global health governance as “the use of formal and informal institutions, rules, and processes by States, intergovernmental organizations, and non-State actors to deal with health challenges that require cross-border collective action to address ‘effectively’” ([20], p.3). Importantly, these enumerated components form interconnected elements comprising a series of partially overlapping clusters, thereby introducing an additional layer of intricacy to global health governance. Each cluster assumes the character of a regime, marked by specific health challenges, strategies, and processes maturing over time, collectively constituting a global health regime complex [20]. We regard this nuanced differentiation as pivotal for cultivating a more profound understanding of the global health governance intrinsic to the 2030 Agenda. Consequently, we curated health-related indicators into thematic groups, systematically exposing them in sequence.

By scrutinizing the VNR indicators as important governance mechanisms of the Agenda, our objective was to assess their presence and alignment with the Global Indicator Framework for the Sustainable Development Goals. This global framework assumes a paramount role, representing a fundamental facet of the Agenda’s overarching objectives of comparability and convergence vis-à-vis preestablished commitments [21]. Our results highlight a concerning discrepancy in the reporting frequency of various health-related themes. This uneven emphasis reveals underlying challenges in the statistical capacity of States and illustrates a broader issue of selective prioritization, which may hinder comprehensive health governance under the SDGs. These insights into VNRs and their operational challenges may offer a nuanced understanding of how global health governance is being shaped. They could contribute to broadening the scope of VNRs as a tool to foster new inquiries and deepen insights into the discussions concerning state narratives on global health governance under the 2030 Agenda and their evident prioritization approaches.

Nevertheless, our approach to studying VNR indicators has important limitations. First, there are limitations related to the development of global SDG indicators, as they are influenced by both political factors and technical issues. This led to noticeable discrepancies between the ambitious goals stated in the 2030 Agenda and the practical indicators used for monitoring. There are significant reductions in the scope and depth of these indicators during their formulation, from the translation of the Rio Declaration into specific goals and targets to their adaptation to the statistical traditions and data availability of different institutions [22]. Moreover, the practical utility of these indicators merits questioning, as their categorizations do not always reflect the complexities of actual health service delivery and experience. Finally, our study does not evaluate the performance of individual countries against each indicator, instead focusing on their presence and alignment within the VNRs. It searches into fostering a critical understanding of how these indicators function, how they appear in VNRs and their effectiveness in advancing global sustainable development.

## Methodology

Our research methodology entailed a qualitative document analysis, executed in two discernible stages. Initially, we meticulously selected and categorized 60 health-related indicators to ascertain their manifestation in the VNRs and evaluate their consistency with the global indicators. The scope of data collection encompassed VNRs documented in English, Spanish, or French and publicized between 2016 and 2020. Specifically, our sample included 20 VNRs in 2016, 42 in 2017, 45 in 2018, 45 in 2019, and 43 in 2020, resulting in a comprehensive total of 195 VNRs. In the subsequent stage, a thorough manual compilation was conducted, encompassing all descriptions of indicators featuring a quantitative outcome in the VNRs, with the exclusion of all references to indicators devoid of statistical results. The amassed data were meticulously cross-referenced against the official global framework of the SDG indicators.

The VNRs and the subsequent compilation of indicators were underpinned by a document analysis method, strategically employed to navigate instances of ambiguity in the VNRs’ presentation of indicators—an aspect that will be explored in greater detail here later. Document analysis, as a qualitative method, serves as a tool for analysis that encapsulates collective memory and history [23]. The methodological approach adopted in this study resonates closely with the tenets of contemporary historiography. It endeavors to interpret documents not only as representations of realities and narratives but also as dynamic entities, acknowledging that archives are not static but rather mutable constructs that can be perceived as institutions. Consequently, archives are conceived as socially constructed within a critical and intellectual framework, giving rise to specific local and temporal claims [24]. Given the limited number of academic studies associated with VNRs, assessments linked to document analysis are predominantly unearthed in the gray literature and internal documents of the UN system. This distinctive context implies divergent interests and institutional objectives [25–29]. Concurrently, alternative initiatives exist, characterized by more quantitative evaluation methods such as benchmarking, text mining, and semiquantitative analysis utilizing ordinal scales. However, it is pertinent to note that these approaches often exhibit a narrow scope [3, 4, 30].

In the initial phase, we employed the categorization of health-related SDG indicators proposed by Silveira et al. (2022), aiming for a comprehensive understanding of health that extends beyond the confines of SDG 3. This methodological approach involved a meticulous review of health-related indicator lists put forth by prominent international institutions: the World Health Organization (WHO), the Pan American Health Organization (PAHO), the World Bank (WB), the Global Burden of Disease (GBD), and the Sustainable Development Solutions Network (SDSN). Through this inclusive strategy, we sought to establish a unified acknowledgement of indicators operationalized by key international entities dedicated to global health and aligned with the objectives of the 2030 Agenda. Consequently, this endeavour identified 60 indicators aligned with the official global indicator framework for SDGs, as developed by the Inter-Agency and Expert Group on SDG Indicators (IAEG-SDGs). These indicators were dispersed across 8 goals and 37 targets, thereby yielding a practical and operationalized compilation of expanded health-related indicators. For a more in-depth elucidation of this methodology, readers are directed to a separate publication [22]. This selection approach was deliberately chosen to surmount the absence of an official definition of health-related indicators. Moreover, in consonance with Fidler’s [20] viewpoint on the impracticality of governing global health under a singular regime, the concept of a regime complex governance housing distinct health regime clusters is analogously applied in this article to categorize health-related indicators according to thematic groups.

For the purpose of categorizing the indicators, we adhered to the WHO classification as delineated in ‘World Health Statistics 2019’. Additionally, we introduced a concluding category labelled “Others” to accommodate indicators operationalized by the other institutions that were noted but not included in the WHO’s health-related list. The selection of the WHO’s categorization stems from its key position as the leading international health authority. The established categories encompass: *(i) Reproductive and maternal health; (ii) Newborn and child health; (iii) Infectious diseases; (iv) Noncommunicable diseases; (v) Injuries and violence; (vi) Environmental risks; (vii) Universal health coverage and health systems; and (viii) Others*. This framework facilitates a targeted examination of indicators with discernible interdependencies, aligning with the WHO convention and offering one of several conceivable thematic divisions within global health regimes and health interventions. Nevertheless, it is imperative to acknowledge that this convention may be perceived as a methodological limitation given the ongoing disputes among health networks concerning the conceptualization of health issues [31] Table 1.

**Table 1:** List of health-related indicators

Thematic groups	Health-related indicators’ themes
(i) Reproductive and maternal health	maternal mortality (3.1.1); birth deliveries (3.1.2); modern methods of family planning (3.7.1); adolescent birth (3.7.2); informed decisions on sexual and reproductive health (5.6.1); laws on sexual and reproductive health care (5.6.2).
(ii) Newborn and child health	stunting (2.2.1); malnutrition (2.2.2); underfive mortality (3.2.1); neonatal mortality (3.2.2); vaccination coverage (3.b.1).
(iii) Infectious diseases	HIV (3.3.1); tuberculosis (3.3.2); malaria (3.3.3); Hepatitis B (3.3.4); NTDs (3.3.5).
(iv) Noncommunicable diseases	mortality to cardiovascular disease, cancer, diabetes, or respiratory disease (3.4.1); suicide (3.4.2); substance abuse treatment coverage (3.5.1); harmful alcohol use (3.5.2); tobacco use (3.a.1).
(v) Injuries and violence	disasters (1.5.1/11.5.1/13.1.1); traffic accidents mortality (3.6.1); intimate partner violences of women (5.2.1); sexual violence of women (5.2.2); child marriage of women (5.3.1); female genital mutilation (5.3.2); occupational injury (8.8.1); homicides (16.1.1); conflict-related deaths (16.1.2); physical, psychological and sexual violence (16.1.3); safety perception (16.1.4); child sexual violence (16.2.3).
(vi) Environmental risks	air pollution (3.9.1); WASH (3.9.2); unintentional poisoning (3.9.3); schools with sanitary services (4.a.1); safe water (6.1.1); safe sanitation (6.2.1); treated wastewater (6.3.1); water and sanitation development assistance (6.a.1); reliance on clean fuels (7.1.2); air quality (11.6.2).
(vii) Universal health coverage and health systems	government spending on essential services (1.a.2); universal health coverage (3.8.1); out-of-pocket health spending (3.8.2); health development assistance (3.b.2); health facilities with medicines available (3.b.3); health workers (3.c.1); IHR capacity (3.d.1); civil registration (17.19.2).
(viii) Others	international poverty line (1.1.1); social protection coverage (1.3.1); undernourishment (2.1.1); access to electricity (7.1.1); migration recruitment cost (10.7.1); migration policies (10.7.2); birth registration (16.9.1); statistical capacity (17.18.1); statistical legislation (17.18.2).

*Source*: Authors

To appraise the alignment with the official global indicators, we meticulously amassed the descriptions of each indicator, specifically focusing on the methodological framework articulated in the textual definition of the indicator. Given that VNRs typically provide minimal additional elements to the indicators, our scrutiny predominantly centered on these text definitions. Each health-related indicator disclosed in the VNR was categorized as exhibiting **‘full consistency’** when it mirrored the global framework verbatim or, if differently worded, was deemed fully encompassed without compromising the integrity of the official metric. An illustrative example of this can be seen in indicator 1.3.1, occasionally presented as “percentage of population covered by social protection”. Indicators were classified as possessing **‘partial consistency’** when they only partially resembled the official indicator or corresponded to a fraction of the requisite phenomenon. This might involve a more confined age group than stipulated in the official indicator, as observed in indicator 3.7.2. Alternatively, ‘partial consistency’ could manifest when the VNR indicator presented different measurements, such as reporting mortality rates instead of incidence rates. The data collection and subsequent analysis of indicator descriptions were meticulously aligned with the official list of the global indicator framework for the SDGs, a repository continually updated by the Inter-Agency and Expert Group on SDG Indicators (IAEG-SDGs). To ensure methodological accuracy, the metrics utilized for contrasting the indicator descriptions corresponded to the versions of the annual refinements published by the IAEG-SDGs each year.

The analytical framework also incorporated the World Bank’s income classification of States in 2020 to discern diverse patterns based on income. The World Bank’s income classification is derived from the Gross National Income (GNI) per capita of each State. Computed using the World Bank Atlas method, this classification delineates four income categories: High-Income ($12,696 or more), Upper-Middle-Income ($4,096 to $12,695), Lower-Middle-Income ($1,046 to $4,095), and Low-Income ($1,045 or less) [32]. This specific categorization was opted for due to its statistical convention, ensuring international comparability, and its notable correlation with nonmonetary indicators assessing quality of life, such as life expectancy and child mortality rates [32].

## Results

Initially, it is imperative to underscore that the process of categorizing the consistency of indicators involves a quantification that should not be divorced from the document analysis method. The heterogeneous characteristics inherent in the production of VNRs play a pivotal role in shaping the observed results. Document analysis assumes a central position in the interpretation of these outcomes, primarily through its content analysis parameters, with a specific focus on historical context, authorship, and the inherent nature of the documents [23]. The examination of the historical context surrounding the 2030 Agenda and global health is indispensable in contextualizing and mitigating interpretations of values espoused within the documents. This approach enables an understanding of the intended audience and the circumstances that precipitated the production of these documents. Our analysis included five distinct years of VNR data, revealing notable heterogeneity in the structure of the reports, particularly in 2016 and 2017. During these initial years, indicators were presented in a more discursive manner and seamlessly integrated into the narrative flow of the VNRs. Subsequent to 2017, there was an update in the guiding guidelines, incorporating more detailed requirements regarding the structure and content expected by the High-Level Political Forum (HLPF) [33]. This refinement contributed to a perceptible trend toward greater standardization in subsequent years.

Furthermore, an examination of the identity, interests, and motivations of the document’s authors is crucial, as is understanding how and where the document is published. Unravelling the identity of the author significantly contributes to assessing the inherent credibility embedded in the document [23]. While the authorship of VNRs is exclusive to the signatory states [34], our scrutiny revealed a diverse array of internal and external institutional actors involved in the national production of these documents. The nature of the document itself aligns with the context of its production, exerting influence on the implicit meanings within the text, the structural composition of the document, and the nuances of authorship. This aspect necessitates a judicious approach in the analysis, acknowledging potential idiosyncrasies within specific sections of the document [23].

The nature of VNRs, being essentially diplomatic, revolves around the national reporting of the implementation of an international agreement. Nonetheless, this study faces significant limitations primarily associated with the constrained role of VNRs as accountability mechanisms and diplomatic documents. The findings are confined to the narratives and partial information encapsulated within the VNRs. Furthermore, a systemic issue within the tradition of national reporting lies in the lack of clarity in government hierarchies regarding the responsibility for preparing reports, often compromising their content [35]. The VNRs exhibit considerable variations in the institutions tasked with coordinating the reporting, encompassing entities such as the Ministry of Foreign Affairs, Presidency, committees, and intersectoral commissions exclusively dedicated to monitoring the SDGs in the States, among others. Each VNR presented individual and contextual governance rationales for the choice of the body responsible for its production. These methodological differences in VNR production also impact the indicators presented.

The results revealed that the overall frequency of health-related indicators in the VNR was 28.7%. In other words, on average, only one-third of the 60 indicators are presented by VNRs. Conversely, we identified a commendable 76% overall average of fully consistent indicators with the official global methodology. However, it is prudent to approach this average with caution, as considerable variations in both frequencies and consistencies were detected within each thematic group. The frequency analysis indicated a temporal increase, with both 2019 and 2020 registering similar numbers. The average frequency between 2016 and 2018 was 22.3%, while the average for 2019 and 2020 increased to 37.5%. Nevertheless, despite this increase in frequency, there was no significant increase in consistency between these two periods. The former period averaged 75.6% full consistency, and the latter period averaged 76.3% full consistency.

Table 2 presents a succinct overview of the findings. In terms of proportional representation, the most frequently referenced thematic groups in the VNRs were *Newborn and child health* at 49.2% and *Infectious diseases* at 38.2%, closely trailed by *Reproductive and maternal health* at 37.2%. In stark contrast, the least frequently mentioned thematic group was *Universal health coverage and health systems* at 16.1%, followed by *Injuries and violence* at 23.2%.

**Table 2:** Consistency of health-related thematic groups according to the official list of indicators (2016–2020)

Thematic groups	Mentions		Full consistency with the official indicators	
	*N*	%	*N*	%
(i) Reproductive and maternal health	435	37.2%	360	82.8%
(ii) Newborn and child health	480	49.2%	362	75.4%
(iii) Infectious diseases	372	38.2%	284	76.3%
(iv) Noncommunicable diseases	259	26.6%	203	78.4%
(v) Injuries and violence	542	23.2%	382	70.5%
(vi) Environmental risks	529	27.1%	360	68.1%
(vii) Universal health coverage and health systems	251	16.1%	188	74.9%
(viii) Others	486	31.2%	410	84.4%
TOTAL	3354	28.7%	2549	76.0%

*Source* Authors

In terms of the consistency of health-related indicators aggregated by thematic groups, the three groups exhibiting the highest levels of full consistency are *Others* at 84.4%, *Reproductive and maternal health* at 82.8%, and *Noncommunicable diseases* at 78.4%. Conversely, the groups demonstrating lower consistency with the official list include *Environmental risks* at 68.1%, *Injuries and violence* at 70.5%, and *Universal health coverage and health systems* at 74.9%.

When scrutinizing the results by income classification, it becomes evident that the two income categories displaying the highest frequency of health-related indicators were Lower-Middle-Income at 34.9% and Upper-Middle-Income at 26.8%. Interestingly, despite constituting almost a third of the total VNRs delivered (29.7%), the High-Income VNRs exhibited the second-lowest percentage of health-related indicators at 21.1%. It surpassed only the Low-Income group, which constituted 13.8% of the VNRs and demonstrated a frequency of 16.8%. In terms of the consistency of indicators with the official methodology, Low-Income States stood out with an impressive 85.7% full consistency. The other income categories hovered around the average of 76% full consistency. In essence, High-Income States exhibited the lowest frequency of health-related indicators, while Lower-Middle-Income and Upper-Middle-Income States displayed the highest frequencies. Notably, Low-Income States distinguished themselves by presenting the global description of the indicator with more consistency compared to other income categories.

Nevertheless, the averages obscure distinct trends within the thematic groups and income categories. Notably, a few indicators significantly influenced the overall averages of those groups. Hence, we find it imperative to present the principal highlights of the results categorized by thematic groups. These highlights are presented in descending order of the highest frequencies.

The *Newborn and Child Health* thematic group emerged with the highest frequency, yet it ranked fifth in terms of full consistency between groups, achieving slightly below the average at 75.4%. Nonetheless, the most frequently mentioned indicators across all income categories were underfive mortality (71.3%) and neonatal mortality (52.3%), both attaining approximately 90% full consistency. Stunting was the sole other indicator exceeding 80% full consistency, even with less than half of the mentions. Notably, indicators related to child malnutrition and vaccination coverage were more prevalent in Lower-Middle-Income and Low-Income States.

The *Infectious Diseases* and *Reproductive and Maternal Health* groups exhibited similar frequencies, with Low-Income States being the most frequent contributors to both groups, followed by Lower Middle-Income States. Frequencies decreased with the increase in income classification, except for reproductive health indicators, which received proportionally low frequencies in all income categories. Notably, the *Infectious Diseases* group displayed the highest consistency rate for tuberculosis (87.3%) and the lowest for HIV (72.9%), despite both indicators having the highest frequency. The Neglected Tropical Diseases (NTDs) indicator recorded the lowest frequency rate at 18.2%. In the *Reproductive and Maternal Health* group, the highest frequency and full consistency rates were observed for maternal mortality (70.8%) and birth deliveries by health personnel (51.8%). Reproductive health indicators of SDG 3 exhibited frequencies close to the group average, but adolescent births had the lowest full consistency rate at 51.9%, while sexual health indicators of SDG 5 were presented in less than a tenth of the VNRs.

The *Others* group garnered the fourth-highest frequency at 31.2% and secured the top spot in terms of full consistency at 81%. Among the most frequent indicators in this group were the international poverty line (82.6%) and access to electricity (62.1%). However, indicators related to migration and statistical legislation achieved frequencies of less than 10%. The social protection indicator recorded the lowest full consistency within the group at 68.9%.

The *Environmental risks* group emerged as the fifth most frequent (27.1%), slightly below the overall average frequency of the groups. Indicators with the highest frequencies included safe access to water (70.3%) and sanitation services (62.1%), while those with the lowest frequencies were mortality from air pollution (8.2%) and water and sanitation development assistance (8.7%). This group exhibited the lowest full consistency (68.1%), and interestingly, the indicators with better frequencies also demonstrated lower full consistencies. Indicators related to more basic sanitation and pollution conditions, such as WASH indicators, schools with sanitary services, safe water and sanitation, and reliance on clean fuels, were predominantly cited by Lower Middle and Low-Income States. On the contrary, indicators focusing on more sophisticated sanitary subjects, such as treated wastewater and air quality, were more frequently cited by Upper-Middle-Income and High-Income States.

The *Noncommunicable diseases* group ranks as the sixth most mentioned (26.6%), with a slightly above-average full consistency. The indicator for cardiovascular disease, cancer, diabetes, and respiratory disease emerged as the most frequent, while the rest of the indicators hovered close to the average. A notable exception in terms of frequency is the indicator for substance abuse treatment coverage, which was among the least frequent (8.2%) in VNRs. The indicator with the highest full consistency was suicide (93.8%), while the indicator with the lowest was substance treatment (33.3%). The exposure of this group varied significantly by income, ascending as States’ incomes increased. It was the least mentioned thematic group by Low-Income States and progressively rose in frequency positions until it reached the top position as the most mentioned group by High-Income States.

The *Injuries and Violence* group stands as the seventh most frequent (23.2%). It features traffic accident mortality and homicides as the most mentioned indicators, both receiving approximately 40% frequency. Conversely, the least mentioned indicators were conflict-related deaths (7.2%) and underage sexual violence (8.2%). Indicators with the lowest full consistencies, approximately 60%, were related to disasters, gender violence, and safety perception. Two discernible trends were identified: one involving occupational injury and homicide indicators, which increase in frequency with higher income classifications, and another involving disaster and gender violence indicators, which increase in frequency with lower income classifications.

Finally, the thematic group ranking last in frequency is *Universal health coverage and health systems* (16.1%). None of the indicators in this thematic group attained a frequency above the general average. However, indicators of health development assistance, health facilities with medicines available, and IHR capacity stand out with a frequency of approximately 5%, placing them among the ten least frequent indicators in VNRs. This group ranks second-to-last in full consistency (74.9%). Indicators with the highest full consistencies were related to health development assistance, health workers, and civil registration, receiving between 80% full consistencies. Notably, there were no significant variations linked to income. Consequently, the group received low frequencies and consistencies across all income classifications. A summary of the main findings and trends of all thematic groups can be found in Table 3 below.

**Table 3:** Summary of thematic findings

Thematic groups	Main trends
(i) Reproductive and maternal health	● 3° Place in citations. ● 2° Place in full consistency to official global framework indicators. ● Low-Income States were the most frequent providers, followed by Lower Middle-Income States. Indicators’ citations decreased proportionally with the increase in the income classification. *Indicators*: ● Highly cited with full consistency: maternal mortality (3.1.1) and birth deliveries by health personnel (3.1.2). ● Low citation: sexual health indicators (5.6.1 and 5.6.2).
(ii) Newborn and child health	● 1° Place in citations. ● 5° Place in full consistency to official global framework indicators: slightly below average. *Indicators*: ● Highly cited with full consistency: underfive mortality (3.2.1) and neonatal mortality (3.2.2). ● Low and Lower Middle-Income States presented considerably more indicators of child malnutrition (2.2.1 and 2.2.2) and vaccination coverage (3.b.1).
(iii) Infectious diseases	● 2° Place in citations. ● 4° Place in full consistency to official global framework indicators: above average. ● Low-Income States were the most frequent providers, followed by Lower Middle-Income States. Indicators’ citations decreased proportionally with the increase in the income classification. *Indicators*: ● Highly cited: HIV (3.3.1) and tuberculosis (3.3.2). ● Low citation: NTDs (3.3.5).
(iv) Noncommunicable diseases	● 6° Place in citations: below average. ● 3° Place in full consistency to official global framework indicators. ● High-Income States were the most frequent providers, followed by Upper Middle-Income States. Indicators’ citations increased proportionally with the increase in the income classification. *Indicators*: ● Highly cited: cardiovascular disease, cancer, diabetes, or respiratory disease (3.4.1). ● Low citation: substance abuse treatment coverage (3.5.1).
(v) Injuries and violence	● 7° Place in citations. ● 7° Place in full consistency to official global framework indicators: second highest partial consistency. *Indicators*: ● Highly cited: traffic accidents mortality (3.6.1) and homicides (16.1.1) ● Low citation: conflict-related deaths (16.1.2) and sexual violence by age 18 (16.2.3). ● Low full consistency: disasters (1.5.1), gender violence (5.2.1, 5.2.2, 5.3.1, 5.3.2) and safety perception (16.1.4). ● Indicators’ citations for occupational injury (8.8.1) and homicide (16.1.1) increased proportionally with the increase in the income classification. Indicators citations for disasters (1.5.1) and gender violence (5.2.1, 5.2.2, 5.3.1, 5.3.2) decreased proportionally with the increase in the income classification.
(vi) Environmental risks	● 5° Place in citations: slightly below average. ● 8° Place in full consistency to official global framework indicators: highest partial consistency. *Indicators*: ● Highly cited: access to water (6.1.1) and sanitation services (6.2.1). ● Low citation: mortality from air pollution (3.9.1) and water and sanitation development assistance (6.a.1). ● Indicators related to more basic sanitation and pollution conditions, such as WASH (3.9.2), schools with sanitary services (4.a.1), safe water and sanitation (6.1.1 and 6.2.1), and reliance on clean fuels (7.1.2) were most cited by the and Low and Lower-Middle -Incomes States. Upper-Middle-Income and High-Income States cited indicators with more refined sanitary subjects, such as treated wastewater (6.3.1) and air quality (11.6.2).
(vii) Universal health coverage and health systems	● 8° Place in citations: near half of the average. ● 6° Place in full consistency to official global framework indicators: third highest partial consistency. ● There were no significant variations linked to income. The group received low frequencies and consistencies across all incomes. *Indicators*: ● Lowest citation: health development assistance (3.b.2), health facilities with medicines available (3.b.3), and IHR capacity (3.d.1).
(viii) Others	● 4° Place in citations: above average. ● 1° Place in full consistency with official global framework indicators. ● Low-Income States were the most frequent providers, followed by Lower Middle-Income States. Indicators’ citations decreased proportionally with the increase in the income classification. *Indicators*: ● Highly cited with full consistency: international poverty line (1.1.1) and access to electricity (7.1.1). ● Low citation: migration recruitment cost (10.7.1); migration policies (10.7.2); statistical legislation (17.18.2). ● Low full consistency: social protection coverage (1.3.1).

*Source* Authors

**Fig. 1:**
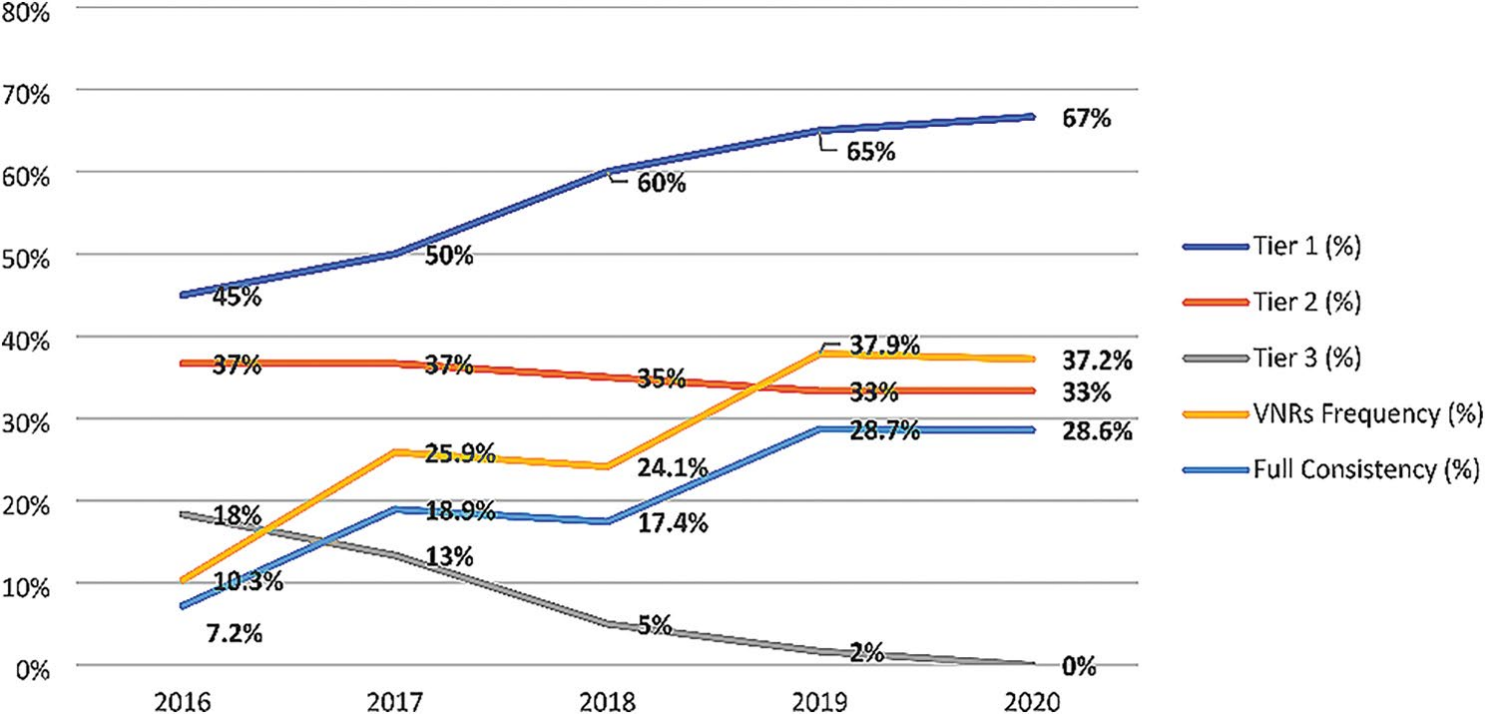
Health-related indicators: Tiers evolution and VNRs (2016–2020)

These results can also be juxtaposed with the evolution of States’ statistical capacity over the years to produce SDG indicators. The IAEG-SDGs monitor the methodological development of the SDGs and the availability of official global indicators in States through a Tiers classification. In this classification, Tier 1 indicates that the indicator is regularly produced by more than half of the countries, Tier 2 indicates that less than half of the countries produce it, and Tier 3 indicates that the indicator still lacks an internationally established methodology or standards [36]. The timeline evolution of the Tiers classifications is illustrated in Fig. 1.

These initial five years provided the IAEG-SDGs with the opportunity to develop the necessary methodological refinements, resulting in the evolution of all indicators from Tier 3. However, by the conclusion of 2020, the IAEG-SDGs acknowledged that, among the 60 health-related indicators selected in this paper, 20 (33%) still held a Tier II methodological classification. This classification indicates that these indicators were not regularly produced by more than half of the signatory States using the algebraic formulas officially requested by the IAEG-SDGs [36]. The failure to achieve regular production for one-third of health-related indicators poses a significant challenge to monitoring capacity and remains a substantial hurdle for governance.

## Discussion

The 2030 Agenda indicators occupy the intersection between science and policy, representing influential elements in complex systems. Even when States, the private sector, and civil society collaborate in developing a shared measurement system, their divergent interests and objectives can lead to varied uses of the indicators [5]. Simultaneously, the absence of an official definition for the operationalization of the 2030 Agenda grants States the flexibility to make their own selections and take actions based on their interpretation [3]. This framework resembles Fidler’s [37] concept of the normative “source code” of global health being applied by States within the governance space of an “open-source anarchy.” In this scenario, States adapt these codes to their unique contexts, and global health governance is shaped by an unstructured plurality [37]. This concept is also analogous to Acharya’s [38] idea of “localization” – the variation in the acceptance and domestication of international norms – and the concept of “translation” commonly used in the literature on the diffusion of public policy [39]. Thus, the utilization of indicators is intertwined with a State’s process of adaptation to the global proposal. The choices of indicators and the degree of consistency presented reflect the diverse profiles of adherence to the agenda and its governance. By examining the confluence of these choices in the first five years, it becomes possible to discern which themes carry greater discursive weight in VNRs.

The initial analysis revolves around the accessibility of the indicators. The official suggestion to employ indicators arose with the anticipation that they would serve as a tool to guarantee the consequential and effective implementation of the proposed targets. This envisioned that the indicators played a key political role within the governance structure of the 2030 Agenda [5]. However, the formulation of indicator definitions was shaped by the interplay of political tensions and disputes among States within the IAEG-SDGs. These disputes have significantly influenced the methodological definition process of numerous indicators for several years [40], as illustrated in Fig. 1.

The challenges faced by half of the States in regularly producing one-third of the health-related indicators, coupled with prolonged political disputes within the IAEG-SDGs that delayed the establishment of official metrics, underscore the inherent struggles of the SDGs in solidifying the official global indicators as effective governance mechanisms for Agenda implementation. Consequently, this directly impacts two crucial elements of SDG governance. First, it compromises the principle of defining global indicators, which plays a pivotal role in facilitating international comparisons of State performance by employing common metrics to gauge the achievement of shared objectives. Second, the use of different proxies has a direct impact on the aspirational nature of the official metrics for various indicators [41, 42]. The proxies presented often represent simplified methodological abstractions of diverse SDG indicators proposed through more intricate or specialized measurements. Paradoxically, this aspirational aspect also renders it more challenging for States’ statistical capacities to collect and process data, thereby affecting SDG governance.

Whether driven by the intent to mitigate this statistical deficit or other motivations, the findings reveal that States opted for numerous proxy indicators to represent the SDG indicators in the VNRs. Therefore, the total number of indicators employed in the VNRs surpasses the expected output of official indicators based on the Tiers classification. While it is challenging to assert an exact correspondence of the results with the methodological classification of indicators in Tiers proposed by IAEG-SDG between 2016 and 2020, this official parameter provides a framework for discussing parallels and contradictions in some results found in the reports. This contributes to elucidating political choices made by States.

The primary findings reveal a concurrent, gradual increase in the presentation of health-related indicators and their full consistency in the VNRs over the years, with notable threefold growth from the initial values in 2016, particularly in the last two years. However, these advancements in indicator reporting should not be considered in isolation. Throughout this period, various governance instruments of the Agenda were actively engaged, including regular reviews of IAEG-SDGs methodologies, ongoing discussions within working groups and with stakeholders, annual HLPF meetings, and recommendations aimed at enhancing the quality of VNRs, among other collaborative efforts.

Nevertheless, it is crucial to highlight that the average representation of indicators in VNRs remains relatively low, with less than one-third of health-related indicators publicly disclosed. This indicates a limited governance role of the VNR as an accountability mechanism for SDG implementation and as an instrument for enforcing SDG governance. Importantly, this scarcity of indicators cannot be solely attributed to the statistical capacity of the States. This becomes evident when we observe a substantial gap, with 67% of the indicators classified as Tier 1, yet only 37% are available in the VNRs for 2020. Hence, additional factors may influence the decision to present these indicators, revealing potential instances of selective reporting that will be elucidated in the following analysis.

Nevertheless, the evident enhancements in the VNRs suggest that States may acknowledge national reporting as a substantial accountability mechanism within the Agenda. Otherwise, they might not invest in improvement, particularly when considering the historical shortcomings of previous international agreements relying on compliance through national reporting [35, 43]. At the very least, it appears to serve as an incentive for participants to enhance and uphold a basic standard of compliance. In line with Young’s assertion [6] that global governance relies on accountability mechanisms, the heightened consistency in presenting indicators can be viewed as a positive stride toward the global governance of the Agenda.

The nuanced question to address is as follows: What are the implications of these results for the Agenda’s global health governance? Despite thematic groupings sharing a common theme, each group encapsulates diverse phenomena, often associated with distinct global health networks. Our hypothesis suggests that indicators within the first four groups - *(i) Reproductive and maternal health, (ii) Newborn and child health, (iii) Infectious diseases*, and *(iv) Noncommunicable diseases* - would exhibit better frequency and consistency. This expectation stems from their association with longstanding issues in public health, historical inclusion in global statistical monitoring, and a likelihood for more uniform presentation across States. On the other hand, the last four thematic groups - *(v) Injuries and violence, (vi) Environmental risks, (vii) Universal health coverage and health systems*, and *(viii) Others* - encompass indicators for newly constructed indicators, or health-related subjects less familiar to health institutions. As a result, these groups exhibit more significant variations and partial consistency with official indicators. While the hypothesis holds true in most cases, counterintuitive results surfaced within the groups, prompting further investigation into their potential motivations.

Moreover, as we contemplate the potential coexistence of strengths and weaknesses and the multifaceted nature of global governance [44], we delve into characteristics associated with both the successes and shortcomings of the 2030 Agenda’s global health governance. While the primary aim is not an exhaustive exploration of each trait individually, certain points of convergence and divergence merit closer examination for their potential implications. This discussion also engages with Shiffman’s [31] comparative study on global health networks to gain insights into these results.

The notable performance of the (i) *Reproductive and maternal health* thematic group, ranking third in frequency and second in full consistency, can be attributed to the exemplary performance of indicators such as maternal mortality (3.1.1) and birth deliveries by health personnel (3.1.2). This aligns with Shiffman’s [31] observation that the global maternal health network is considered a high global priority, supported by robust coalitions, funding mechanisms, and governance structures. The presence of these indicators in the Millennium Development Goals (MDGs) further facilitated their prominence. However, it is crucial to recognize that having a strong network does not guarantee methodological consistency, as evidenced by the adolescent birth indicator (3.7.2), which exhibited the lowest full consistency. This inconsistency arises primarily from the lack of stratified data for girls aged 10–14 years, highlighting a significant data deficit that has spurred calls for urgent action [45]. On the other hand, indicators related to reproductive and sexual health, particularly those within SDG 5 on gender equality (3.7.1, 5.6.1, and 5.6.2), displayed subpar performance. The IAEG-SDGs acknowledge a low global production of these indicators, with indicator 5.6.2’s methodology defined only after 2018 [46, 47]. However, evidence suggests that certain statistical deficits are tied to global stigma, policy choices, and inadequate public investment in sexual and reproductive rights topics [48, 49].

In the case of the (ii) *Newborn and child health* group, attaining the highest frequency of mention is unsurprising, given that the IAEG-SDGs acknowledged all indicators in 2018 as regularly produced in the official methodology by more than half of the states (Tier I). The IAEG-SDGs emphasize that child indicators benefit from an international statistical tradition, facilitating their data collection and production [50, 51]. However, the challenge lies in the group’s fifth rank in terms of full consistency (75.4%) between groups, which is slightly below the average. If the data are readily accessible, why resort to numerous proxies? One explanation might be the substantially greater mention of indicators by Lower-Middle-Income and Low-Income States coupled with their comparatively lower statistical capacity, leading to an increased reliance on proxies. Notably, the most mentioned indicators in these income categories were child malnutrition (2.2.1 and 2.2.2) and vaccination coverage (3.b.1). However, Shiffman’s [31] observation that the ‘Early Childhood Development’ global health network is fragmented, with divergent interests among stakeholders, might offer additional insights into this phenomenon. The presence of proxies may signify not only data accessibility challenges but also underlying complexities within the global health network focused on early childhood development.

The second most frequently addressed thematic group, (iii) *infectious diseases*, is also ranked fourth in terms of full consistency, closely approaching the overall average. Yet, this average consistency diverges from the evaluation by the IAEG-SDG, which is the only other thematic group to have all indicators classified as Tier I since 2018 [36]. The notable prevalence of high frequencies for the HIV (3.3.1) and tuberculosis (3.3.2) indicators aligns with the perceived robustness of governance and funding prioritization within global health networks dedicated to these two phenomena [31, 52]. Nevertheless, the strength observed in these indicators does not rationalize that the HIV indicator has a notably lower percentage of full consistency. This discrepancy arises because this indicator is frequently presented with population data rather than adhering to the requisite framework of the incidence rate of “new HIV infections,” data that the IAEG-SDGs assert are readily available. Another perplexing aspect is the Neglected Tropical Diseases (NTD) indicator (3.3.5), which exhibits half the average percentage and low full consistency, despite being classified as Tier I since 2016 by the IAEG-SDGs. This classification raises questions, especially given the IAEG-SDGs’ report that 191 states individually account for NTDs, with the majority not producing this aggregated indicator [53]. Beyond the outcomes themselves, this recognition prompts an inquiry into the parameters employed by the IAEG-SDGs for tier classification. Does it hinge solely on regular indicator production, or does it also consider the availability of resources required for production? The implications of this distinction for the governance of SDG indicators warrant careful consideration.

Contrary to our initial conjecture, the (iv) *Noncommunicable Diseases* group did not attain a high frequency of mentions and ranked second to last among the thematic groups, falling below the average. However, it is notable that the group demonstrated greater full consistency, positioning it in third place among the thematic groups. A plausible explanation for this outcome could be that High-Income States mentioned these indicators more frequently, given their superior statistical capacity, funding, and prioritization of this theme due to demographic transitions and the heightened burden on their health systems [54, 55]. However, the decreasing frequency with decreasing income levels poses an additional challenge to the global governance of this group. Undoubtedly, there exists an escalating “double burden” of infectious and noncommunicable diseases for the health systems of Lower-Middle-Income and Low-Income States in the 21st century [54]. If the issues persist, one might speculate whether their preference to highlight the *Infectious diseases* group over the *Noncommunicable Diseases* group signifies policy prioritization or if the impacts of noncommunicable diseases are yet to be comprehensively addressed in their national health and statistical systems. Moreover, despite Shiffman’s [31] observation of relatively robust global tobacco and alcohol control networks, this did not manifest in enhanced results regarding the mention or consistency of their indicators in VNRs. Last, the subpar result for coverage of treatment interventions (3.5.1) may be linked to the IAEG-SDGs’ delayed definition of its official methodology, occurring only in 2019. Even if proxies exist, the following question arises: what urgency is there for States to report an indicator that the IAEG-SDGs themselves have not prioritized defining?

The (v) *Injuries and Violence* group is ranked seventh in both frequency and full consistency. Even the most prominently featured phenomena, such as traffic fatalities (3.6.1) and homicides (16.1.1), were only slightly above the overall average. The IAEG-SDGs acknowledge the persistent challenge of low production for this group of indicators, with only four (1.5.1, 3.6.1, 5.3.1, and 5.3.2) classified as Tier I after the 2020 Comprehensive Review [36]. However, their frequency in VNRs does not reach half of the number of States. Addressing violence and injury issues within health governance is intricate due to their association with a myriad of underlying causes, including social and cultural norms supporting violence, gender disparities, social and economic inequalities, as well as the misuse of alcohol, drugs, and firearms [52, 56]. Notably, among the 12 indicators in this thematic group, indicator 3.6.1 is the only indicator with the World Health Organization (WHO) as the international agency responsible for global monitoring. The other agencies monitoring the remaining indicators in this thematic group are the United Nations Population Fund (UNFPA), the United Nations Children’s Fund (UNICEF), and the United Nations Office on Drugs and Crime [57]. These organizations participate in global networks and adopt distinct approaches to address these phenomena. The ongoing debate on strengthening governance related to these issues necessitates comprehensive multisectoral approaches that effectively target their underlying causes [52].

The (vi) *Environmental Risks* group ranks fifth in frequency but holds the last place in full consistency. Although slightly below the overall average, this group distinguishes itself by being the best positioned among the last groups (*v, vi, vii*, and *viii*), sharing indicators with less statistical tradition in global health. Four indicators within the group reveal a substantial gap between their mention frequency in VNRs and their corresponding rankings in the IAEG-SDGs Tiers. Specifically, indicators for drinking water (6.1.1) and sanitation services (6.2.1) fall under Tier II and are among the ten most frequently cited across all VNRs. Notably, both indicators were relevant under the Millennium Development Goals (MDGs). In contrast, mortality from air pollution (3.9.1) and water and sanitation development assistance (6.a.1) are Tier I and among the ten least cited overall in the VNRs. Income significantly influences contributions to group frequencies, with Lower-Middle and Low-Income States citing indicators related to more basic sanitation and pollution conditions (3.9.2, 4.a.1, 6.1.1, 6.2.1, 7.1.2). Conversely, indicators 6.3.1 and 11.6.2, requiring better sanitation infrastructure, more refined methodologies, and greater statistical capability [58, 59], were cited most by Upper-Middle-Income and High-Income States. Despite their low frequency and consistency, all indicators in the group are under the World Health Organization’s (WHO) custodianship, indicating a potentially greater degree of ownership by global health governance than the indicators related to *Injury and Violence*.

The (vii) *Universal Health Coverage and Health Systems* group attained the lowest frequency, almost half the overall average, and the third-worst consistency. The scant representation of this topic and its position as the least exposed group in the VNRs cannot be entirely justified by statistical capability, given that the IAEG-SDG stipulates that six indicators should be produced by at least half of the states as of 2018 (Tier I). Remarkably, the Coverage of Essential Health Services indicator (3.8.1), computed as the WHO’s Universal Health Coverage (UHC) index, exhibited only a 26.2% frequency in the VNRs, despite being in Tier I since 2018. This raises concerns regarding the WHO’s 69th World Health Assembly resolution in 2016, which aimed to track progress towards achieving UHC as part of the SDGs [60]. Regarding income, a contradictory pattern emerges, as High-Income States show the lowest averages in this group, despite possessing superior statistical capabilities and means of financing health systems [56]. This contradiction also challenges the performance of High-Income States, which generally have significantly higher service coverage than Low- and Middle-Income States. However, it is worth noting that the global improvement in the UHC index from 45 in 2000 to 66 in 2017 is attributed to the most substantial performance increase from Low- and Middle-Income States [61]. This suggests that these States might be more motivated to publicize their progress, creating a logic wherein there is more to gain by sharing advancements in the international arena. This rationale may also be applicable to the results of other thematic groups with a higher frequency of indicators from those States.

The outcomes of the VNRs suggest that States accord less significance to health services monitoring, aligning with the scholarly argument that indicators related to health systems have been insufficiently examined due to the predominant “vertical” approach to health interventions in global health [62]. This vertical orientation is intertwined with the establishment of specialized global health networks focusing on particular health phenomena and conditions, thereby fostering fragmentation and inadequacy of funding for the governance of both global and national health systems [31]. Challenges in data collection for this subject often stem from disjointed systems, lack of standardization, suboptimal data quality, and constrained analytical capacity [52].

The global governance of health coverage and health systems faces constraints due to the predominant political and financial responsibility of states over the infrastructure of health systems [37]. A potential remedy to mitigate this dependence on States for global governance lies in heightened external funding from institutional mechanisms associated with the 2030 Agenda and stakeholders aimed at enhancing the statistical capacities of developing states to implement the SDGs. Although this prospect was deliberated in the early stages of constructing the Agenda, the political disputes within the IAEG-SDGs impeded further discussions and precluded the determination of the source of external funding for the Agenda [40].

Last, the (*viii*) *Other group*, characterized by differences in the nature of the phenomena it encompasses, employs the term “thematic group” cautiously due to its inclusion being necessitated by events not covered in the WHO methodology but acknowledged as directly linked to health by other prominent international organizations in the realm of global health (PAHO, World Bank, GBD, and SDSN). This group secured the fourth position in terms of frequency and claimed the first spot in full consistency, primarily driven by the fact that the most frequently cited indicators also exhibited the highest consistency, elevating their overall average. The findings indicate that States were inclined to highlight phenomena related to poverty (1.1.1) and access to energy (7.1.1), both of which were integral to the MDGs. Simultaneously, states opted for minimal reporting on phenomena such as migration (10.7.1 and 10.7.2) and the international regulation of statistical capacity (17.18.1).

Within the (*viii*) *Other* group, the notable absence of health-related migration in the VNRs is a noteworthy observation. Migration has increasingly emerged as a topic of discussion in global health. Recent studies within the global health domain have delved into the connection between the health conditions of immigrants and health systems, recognizing this population’s heightened vulnerability and precarious living conditions associated with poverty, social exclusion, and discrimination [63]. Health organizations emphasize how immigration policies can impose additional stress on health systems and restrict access to healthcare. From a global health perspective, there is an argument that immigration should be viewed as a social determinant of health [64]. Despite its escalating relevance in global health discourse, the delay in the official methodological definition by the IAEG-SDGs might be linked to the inherent methodological challenges in studying migrant populations, such as the lack of records for illegal immigrants and their national statistical invisibility [63]. On the other hand, the indicators related to migration being among the least cited in the VNRs could be attributed to the political sensitivity of the issue and the explicit exclusion of immigrants from public policies and the distribution of government resources in several states [64].

The variations in the selection of health indicators by States can be attributed to several factors. First, these discrepancies may be tied to the numerous scientific and technical challenges inherent in the 2030 Agenda [65, 66]. Methodological issues in defining globally harmonized indicators and a dearth of national data have posed significant hurdles [40, 41]. Numerous authors have highlighted the technical and financial limitations of States’ statistical capacities, encompassing a lack of disaggregated data and monitoring capabilities [7]. Inadequacies in knowledge and capacity related to systems analysis and integrated planning of public policies, coupled with the absence of an effective structure for prioritizing goals and technical training tailored to the SDGs, further contribute to these challenges [3].

The challenges associated with capacity building represent a significant impediment to global health governance, given that public health infrastructure and policies fall within the purview of States’ political and financial responsibilities [37]. Additionally, while the conventional approach for advancing global health advocates for the development of resilient health systems, this perspective tends to overlook the root causes of existing health inequalities and stresses [12].

Furthermore, political factors play a crucial role in shaping global health networks and are influenced by economic crises, State interests, and the incorporation of health issues into overarching global goals such as the SDGs [31]. The VNRs not only serve as an accountability mechanism for the Agenda but also function as diplomatic documents. Historical instances in global health illustrate that foreign relations within the health sector can be driven by motives beyond the pursuit of health outcomes, including economic, diplomatic, and strategic considerations [67]. For instance, during the MDGs, States often selectively chose indicators that highlighted success while overlooking those that exposed weaknesses or challenges [68]. In this context, VNRs have become increasingly utilized in performance evaluations to showcase political priority-setting [69]. Regarding variations in the selection of SDGs, evidence suggests that States make strategic choices based on the perceived importance of specific SDGs in a strategic hierarchy [70], driven by political expediency, or reflecting a lack of commitment to international agreements [3].

These political factors may lead to harmful goal prioritization, which within the framework of the SDGs occurs when States cherry-pick SDGs that align more closely with immediate economic or political benefits or with their prior development plans, sidelining equally crucial goals that might address more pressing sustainable or ethical challenges [4]. Moreover, the way the SDGs are framed in policy can create a hierarchy in which some goals are perceived as more fundamental or urgent than others, influenced by political framing rather than objective needs assessments [71]. Often, there is a disproportionate emphasis on economic growth over sustainable resource use, illustrating a prevalent global prioritization trend that can perpetuate environmental degradation and inequality [72]. The persistent practice of harmful goal prioritization creates a hierarchy of goals that undermines the holistic and integrative approach of the 2030 Agenda and poses an ongoing challenge to SDG global governance [4]. Adopting a selective focus on only a few priorities within a goal-setting framework may divert attention from other SDGs and compromise the overarching principles of the Agenda. This approach can generate perverse incentives and hinder the implementation of alternative public policies, given the limitations of available financial, technical, and human resources [73]. It is important to recognize the interdependencies between SDGs in policymaking to avoid such harmful prioritizations. A nuanced understanding of these interconnections can lead to more coherent and effective policy advice that supports a balanced pursuit of all goals [74].

The repeated pattern of High-Income States consistently exhibiting the lowest average exposure across several thematic groups traditionally associated with health raises intriguing questions about the underlying political motivations. Given that High-Income States possess superior statistical capabilities and financial means for health system financing [56], one may ponder the rationale behind this behavior. Is it indicative of a lack of prioritization, suggesting that these States consider the targets associated with these indicators already achieved and, therefore, unnecessary to highlight? Does it reflect a belief that continuous monitoring is unwarranted, or is there a potential risk of exacerbating problems through invisibility? Alternatively, could it be an attempt to conceal existing shortcomings in their systems that might contradict their international image? These questions underscore the complexities and potential political dimensions surrounding the reporting choices of High-Income States.

The decision of High-Income States to feature a limited number of health-related indicators in the VNRs prompts contemplation of its implications for the global health governance of the Agenda. Does this signify a disregard for the VNR reporting tool, or does it suggest a shift in thematic investments? Could the sparse mentions of indicators be attributed to limitations in the governance framework of the Agenda, or does it indicate a lack of interest on the part of these states? Are High-Income States in such a favourable international position that they feel exempt from demonstrating accountability through the VNRs? These questions underscore the need for a nuanced analysis of the motives and consequences of the reporting choices made by High-Income States within the context of global health governance.

The commencement of SDG implementation has revealed the political dimensions of indicators, highlighting their usage and relevance to public policies. In light of these factors, it becomes crucial to comprehend the interaction between the formulation of indicators and the policies outlined by the SDGs [5]. Additionally, addressing aspects of the SDGs that lack widespread consensus, both causally and normatively, poses a substantial challenge for meaningful action—an issue that has historically complicated global sustainability governance endeavors [75].

Moreover, the discussion about the low utilization of indicators in the VNRs does not necessarily imply that the performance of the indicators presented surpasses those omitted in achieving the targets. The WHO acknowledged significant global health improvements prior to the COVID-19 pandemic. Interestingly, the indicators demonstrating the most substantial improvements were related to maternal, perinatal, and nutritional conditions, along with various communicable and noncommunicable diseases [61], aligning with the groups of indicators most frequently featured in the VNRs. However, establishing a correlation between the superior performance of indicators and their discretionary selection in the VNRs would necessitate a more intricate analysis involving an investigation into each State’s performance and the corresponding internal debates surrounding VNR creation. Another point of discussion is the UN Secretary-General’s observation on SDG monitoring, which highlights a “continued unevenness of progress”, resonating with the significant variations in the frequency and consistency of indicators across thematic groups and income categories in the VNRs. A more in-depth study is required to comprehend the underlying reasons and consequences of these variations.

## Conclusions

The improvements in the narratives of VNRs signify an increasing commitment by States to report progress on the 2030 Agenda following the UN standard. This commitment, as reflected in our results, manifests in both quantity and quality, demonstrating consistent improvement over the initial five years. However, our analysis reveals a disconcerting trend: despite the apparent progress in reporting on the 2030 Agenda, the engagement of States remains inherently flawed. This flawed engagement is not merely a cause for concern; it represents a significant impediment to the genuine realization of the Agenda’s holistic goals. Our findings indicate that this engagement is characterized by selectivity and heavily influenced by national interests, capacities, and specific challenges. This selective approach undermines the overarching ambition of the 2030 Agenda’s global plan of action, raising questions about critical issues such as harmful goal prioritization and statistical capacities that must be addressed to fulfil this very ambition.

Our findings uncover a noteworthy upwards trend in the inclusion of health-related indicators in VNRs over the years, accompanied by an increased alignment with the official methodology. This trend was especially pronounced in 2019 and 2020. The observed progress serves as a positive indication that the efficacy of 2030 Agenda governance hinges on the adaptability of its institutional mechanisms and its flexibility to sustain States’ engagement. Moreover, our results emphasize the pivotal role of VNRs as reporting mechanisms that integrate both short- and long-term feedback, highlighting their significance in fostering an adaptive approach.

Nonetheless, this apparent improvement is a more profound concern. In the ‘best’ years, less than 40% of health-related indicators were reported. This striking shortfall raises serious doubts about the efficacy of VNRs as instruments for accountability and comparability, thereby diminishing the capacity of these indicators to serve as effective tools for monitoring and evaluating public policies.

Our findings underscore the inherent challenges within the existing SDG indicator framework. A notable discrepancy exists in the reporting of different health-related themes, with several challenges tied to the frequency and consistency of indicators arising from the statistical capacity of States. Notably, there is a discernible pattern wherein indicators with relatively high availability of underlying data are more frequent, a trend observed particularly in thematic groups such as *Reproductive and maternal health*, *Newborn and child health*, and *Infectious diseases*. These groups align with phenomena that boast a lengthy history of global monitoring predating the SDGs. In contrast, thematic groups such as *Injuries and violence* and *Environmental risks*, which exhibit lower frequency, are characterized by a greater concentration of indicators featuring new global methodologies proposed by the IAEG-SDGs, resulting in diminished underlying data availability. The group with the lowest frequency, *Universal health coverage and health systems*, stands out due to its internationally standardized yet underused indicators in global health. This phenomenon can be attributed to the prevailing “vertical” perspective that prioritizes monitoring specific health phenomena and interventions over comprehensive health systems. Unlike other groups displaying variations based on income, this group was overlooked across all income levels.

The discretionary nature of indicator selection by States in the VNRs, enabled by the Agenda’s proposition of a contextual adaptation of the SDGs and a blind eye to the HLPF’s guideline to review all SDG indicators, highlights a critical flaw in the VNR as an accountability mechanism. This becomes particularly evident in the low frequency of certain indicators, despite their reported production by more than half of the States. Such a discrepancy suggests a selective prioritization of themes, driven more by political will than by a steadfast commitment to comprehensive health governance. In terms of State prioritizations based on the presented frequencies, the thematic groups that emerged as the most prioritized were *newborn and child health, infectious diseases, and reproductive and maternal health*. This prioritization is notably pronounced due to the heightened exposure of indicators from lower-middle-income and low-income states. In contrast, High-Income States exhibited the fewest health-related indicators, emphasizing their preference for the *Noncommunicable Diseases* and *Environmental Risks* groups. The *Injuries and Violence* and *Universal Health Coverage and Health Systems* groups could be considered the least prioritized, particularly the latter. These prioritizations serve as indicators of the strengths and weaknesses in the global health governance of the SDGs and may assist in identifying instances of harmful goal prioritization.

The VNRs indicate a potential correlation between statistical capacities and political preferences, contributing to the paradox of simultaneous growth in technical capacity and low governance. This underscores a significant gap between global health governance thematic indicators and their consistency. The mixed results align with Zurn’s conception, suggesting a parallel coexistence of strengthening and declining global governance trends within various spheres of the 2030 Agenda. This indicates diverse governance trends and approaches within the broader context of global health, reflecting disputes, diverse network structures, and overlapping health regime clusters. However, it is crucial to further investigate this evidence at the implementation level within individual States to establish a meaningful comparison between the discursive content of the VNRs and the actual progress achieved.

The narratives within the VNRs strive to align positively with the Agenda’s vision of a more interdependent concept of health. However, incongruities persist, particularly in the use of proxy indicators rather than official global indicators. The frequent use of proxy indicators requires closer examination. Although this may be understandable given the novelty of some official indicators and the ongoing methodological discussions, relying on proxies often involves simplifying the intended scope of the official indicator. This simplification results in a significant loss of nuanced data, contradicting the 2030 Agenda’s ambition for innovative statistical measurements of complex issues. Moreover, the excessive use of proxies may mask a tendency to strategically report, avoiding potential embarrassment associated with unfavourable results. These incongruities ultimately compromise the desired comparability of data and challenge the effectiveness of the global governance of SDG indicators.

Given these findings, it is crucial to recognize that the current state of VNRs and the global indicators framework fall short of their potential as effective tools for global health governance under the SDGs. These discrepancies must be acknowledged and thoroughly investigated to enhance the global health governance of the SDG framework. Considering the 2030 Agenda’s health aspect as a regime complex governance, we propose further exploration of each health regime cluster to gain a better understanding of the elements involved in contention and to discern patterns and State preferences on specific themes. Such an approach allows for maintaining health interdependence with other SDGs while respecting the unique characteristics of distinct health issues. Policymakers and researchers should also address the detrimental prioritization of health-related indicators to uphold the interdependency of health issues, uphold the Agenda’s principles, and facilitate integrative public policies.

On the flip side, it is crucial to recognize that States’ selection and presentation of indicators do not necessarily align with the actual implementation of the SDGs. Therefore, our findings should not be employed to analyse the internalization process and implementation of the SDGs. However, they can contribute to broadening the scope of VNRs as an instrument to introduce new questions and insights into the debates surrounding States’ narratives on the global health governance of the 2030 Agenda and their perceived prioritization.

However, monitoring and review cannot rely solely on the reporting of the VNRs and the HLPF. It needs to be integrated into a more comprehensive system that guarantees more substantial forms of accountability. Without the support of active governance, there are concerns regarding the ability of institutional arrangements to effectively contribute to the implementation of the SDGs. A more comprehensive system that ensures greater accountability and active governance is urgently needed. Without this, these institutional arrangements risk losing legitimacy and effectiveness in achieving accountability for the SDG targets and the Agenda’s implementation.

## Abbreviations

GBD: Global Burden of Disease
HLPF: High-level Political Forum on Sustainable Development
IAEG: SDGs-Inter-agency and Expert Group on SDG Indicators
MDGs: Millennium Development Goals
NTDs: Neglected Tropical Diseases
PAHO: Pan American Health Organization
SDG: Sustainable Development Goals
SDSN: Sustainable Development Solutions Network
UNFPA: United Nations Population Fund
UNICEF: United Nations Children’s Fund
VNR: Voluntary National Reviews
WB: World Bank
WHO: World Health Organization
